# Supplementary figures and images for: Molecular phylogeny and species delimitation of the freshwater prawn Macrobrachium pilimanus species group, with descriptions of three new species from Thailand
Source: PeerJ. 2020 Nov 27;8:e10137. doi: 10.7717/peerj.10137 (PMC7703394; doi:10.7717/peerj.10137)

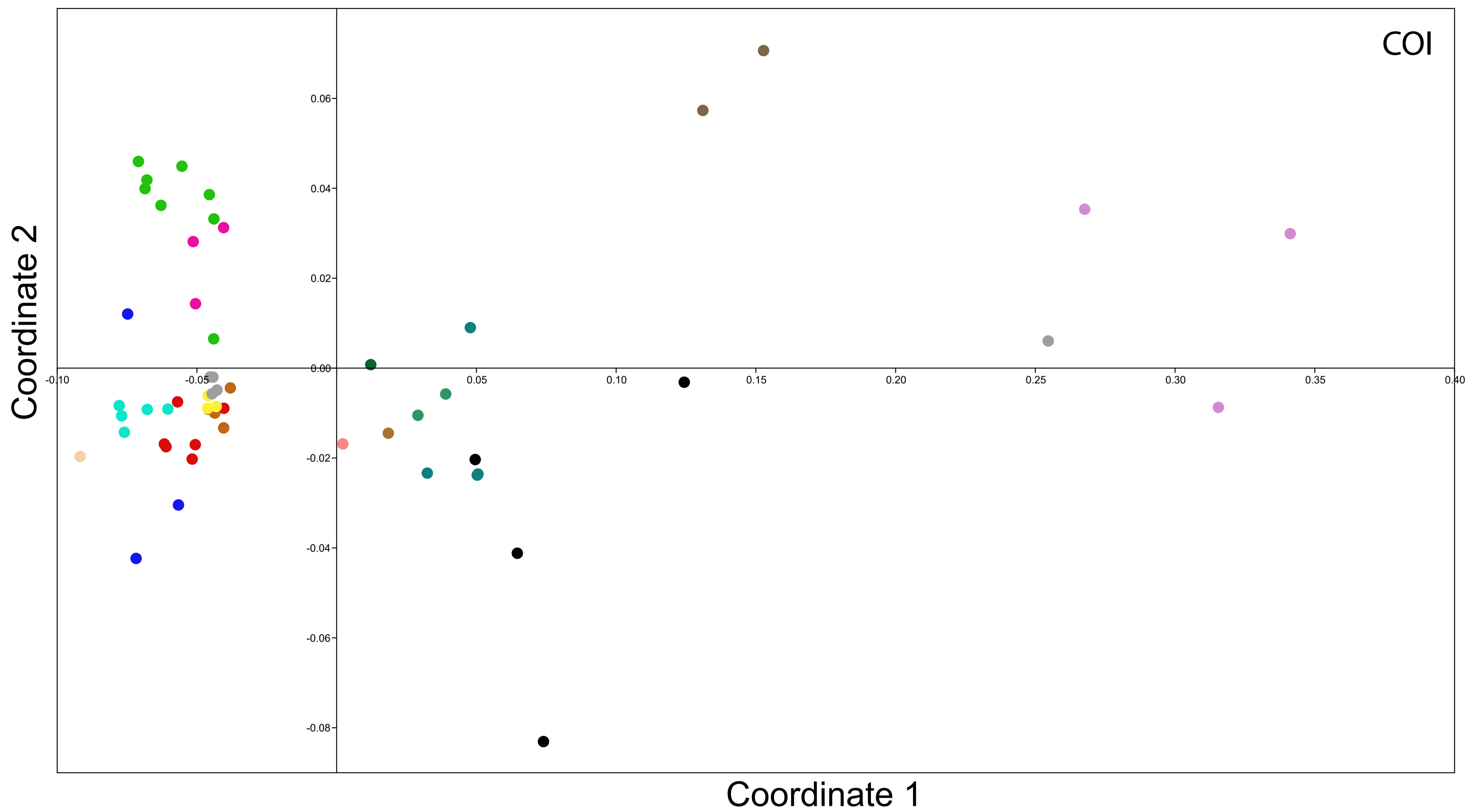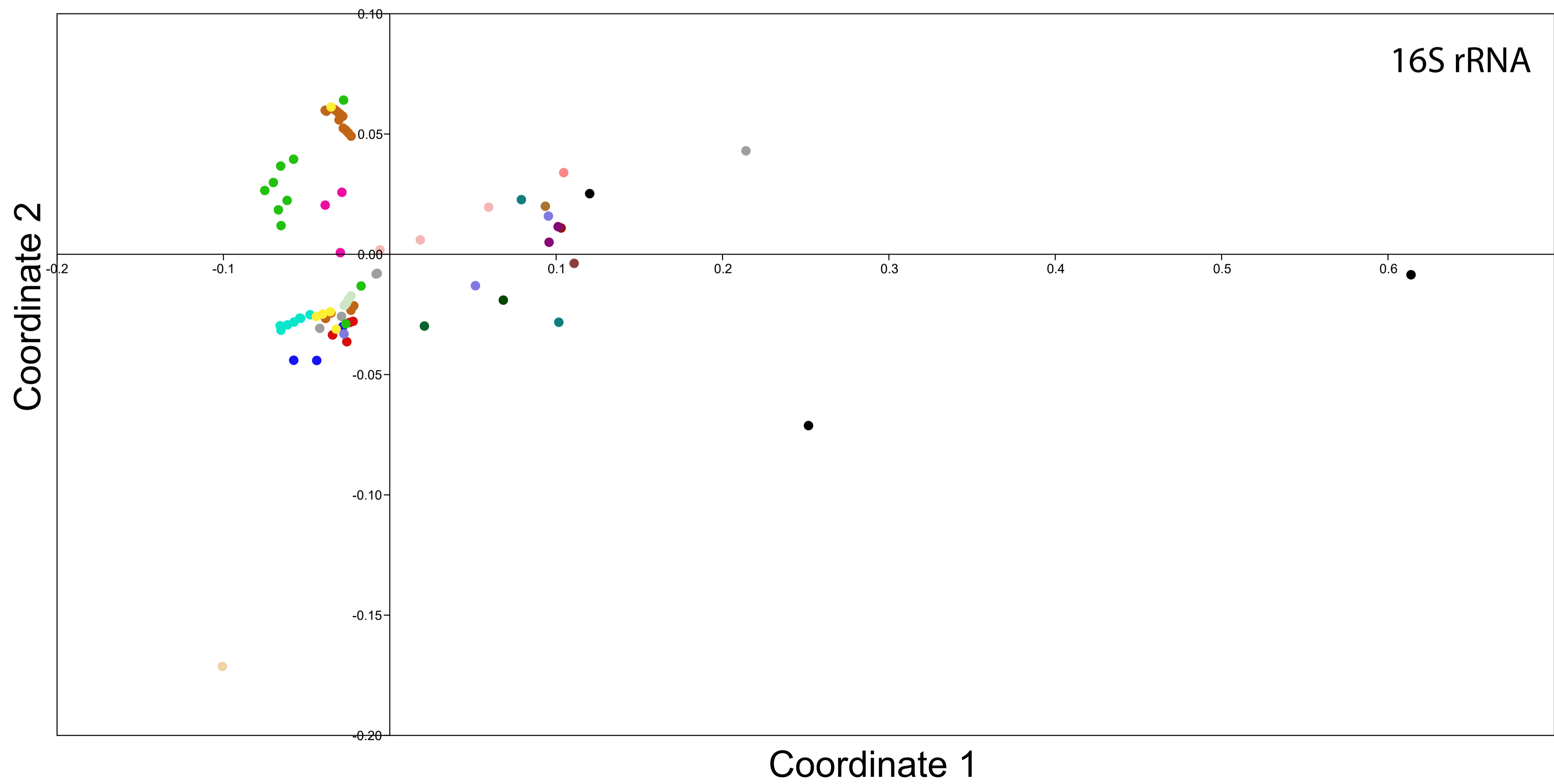

Supplement: Figure S1 [file peerj-08-10137-s001.pdf]

COI

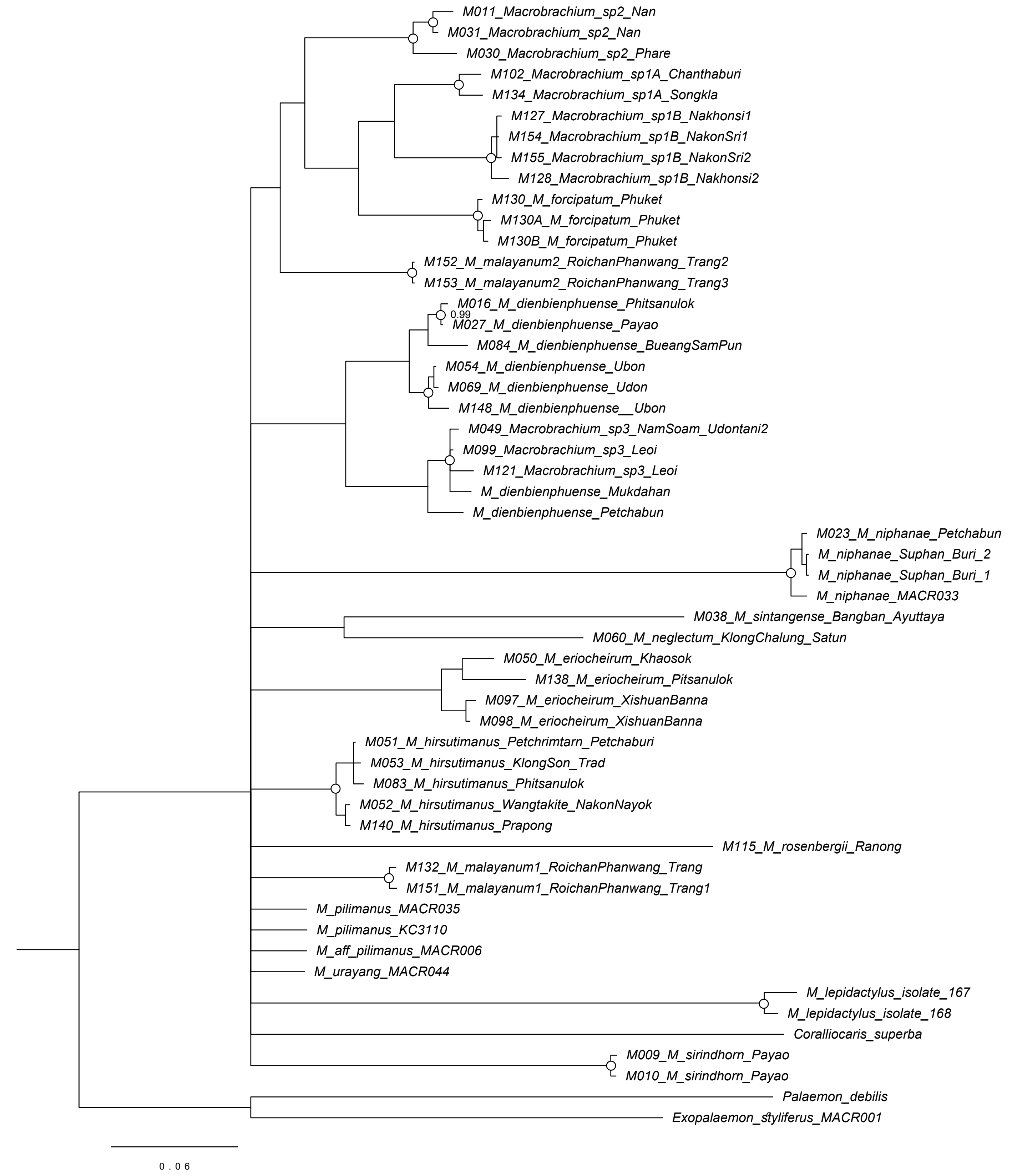

16S

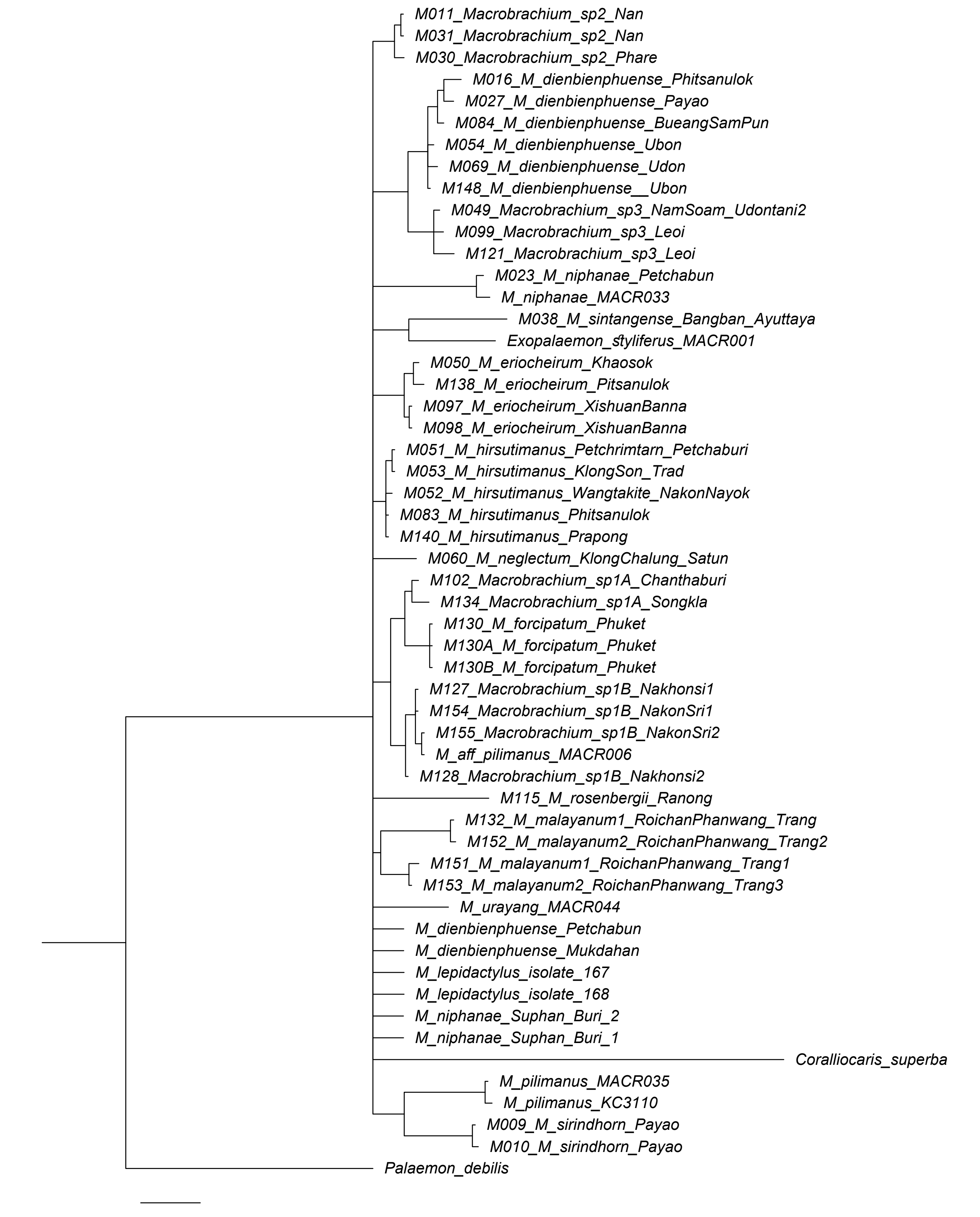

18S

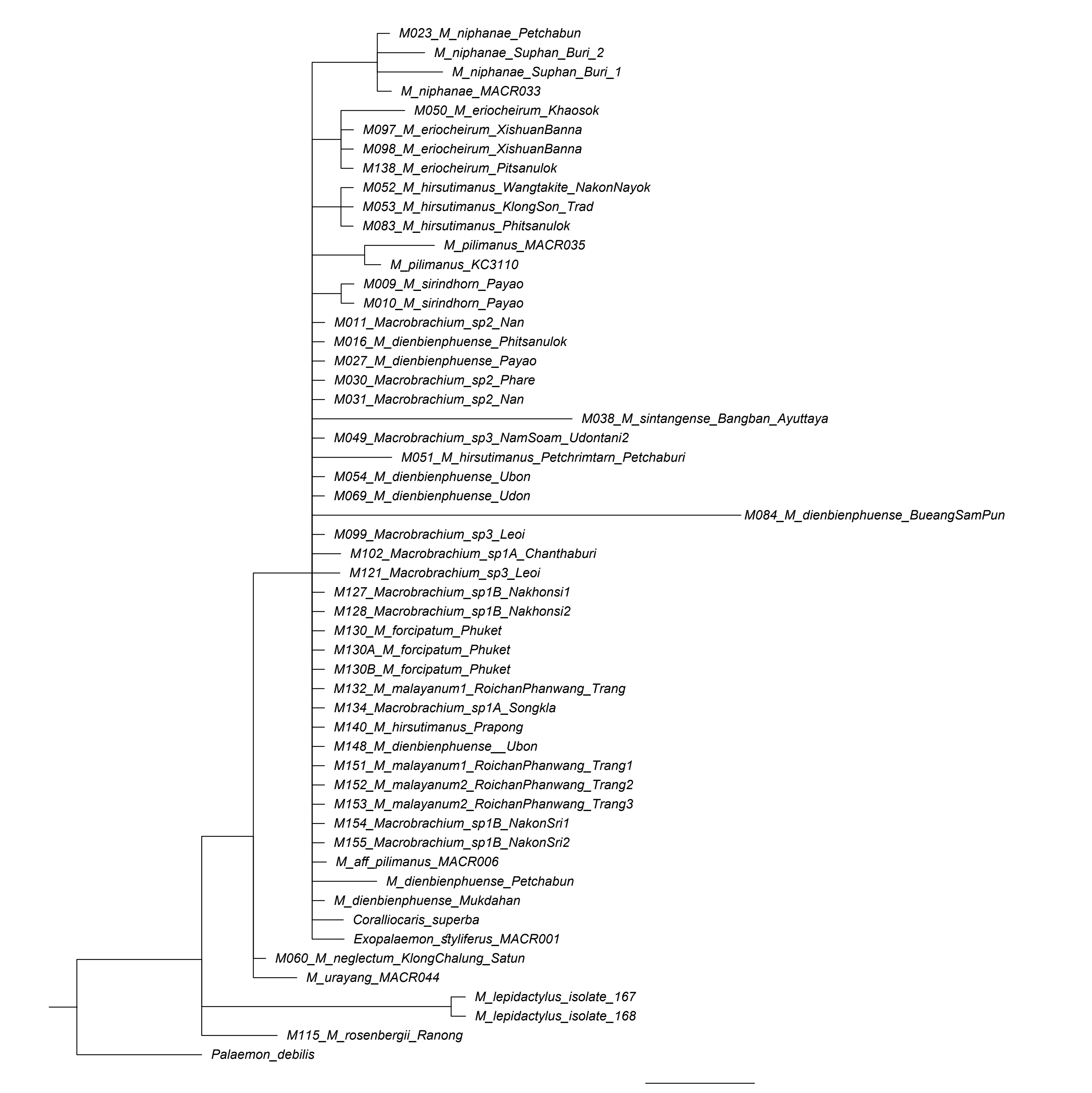

Supplement: Figure S3 [file peerj-08-10137-s003.pdf]

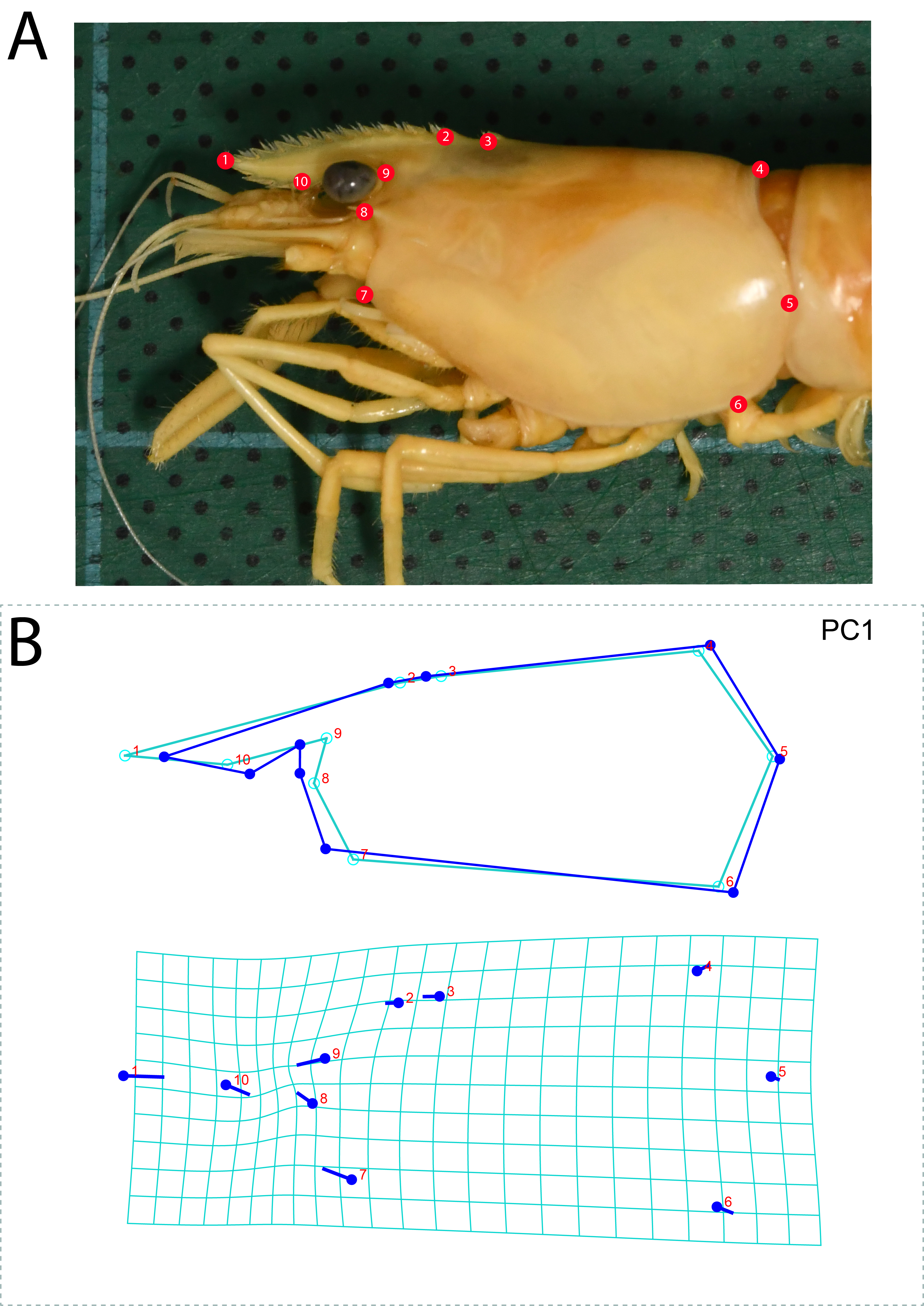

Supplement: Figure S4 [file peerj-08-10137-s004.jpg]

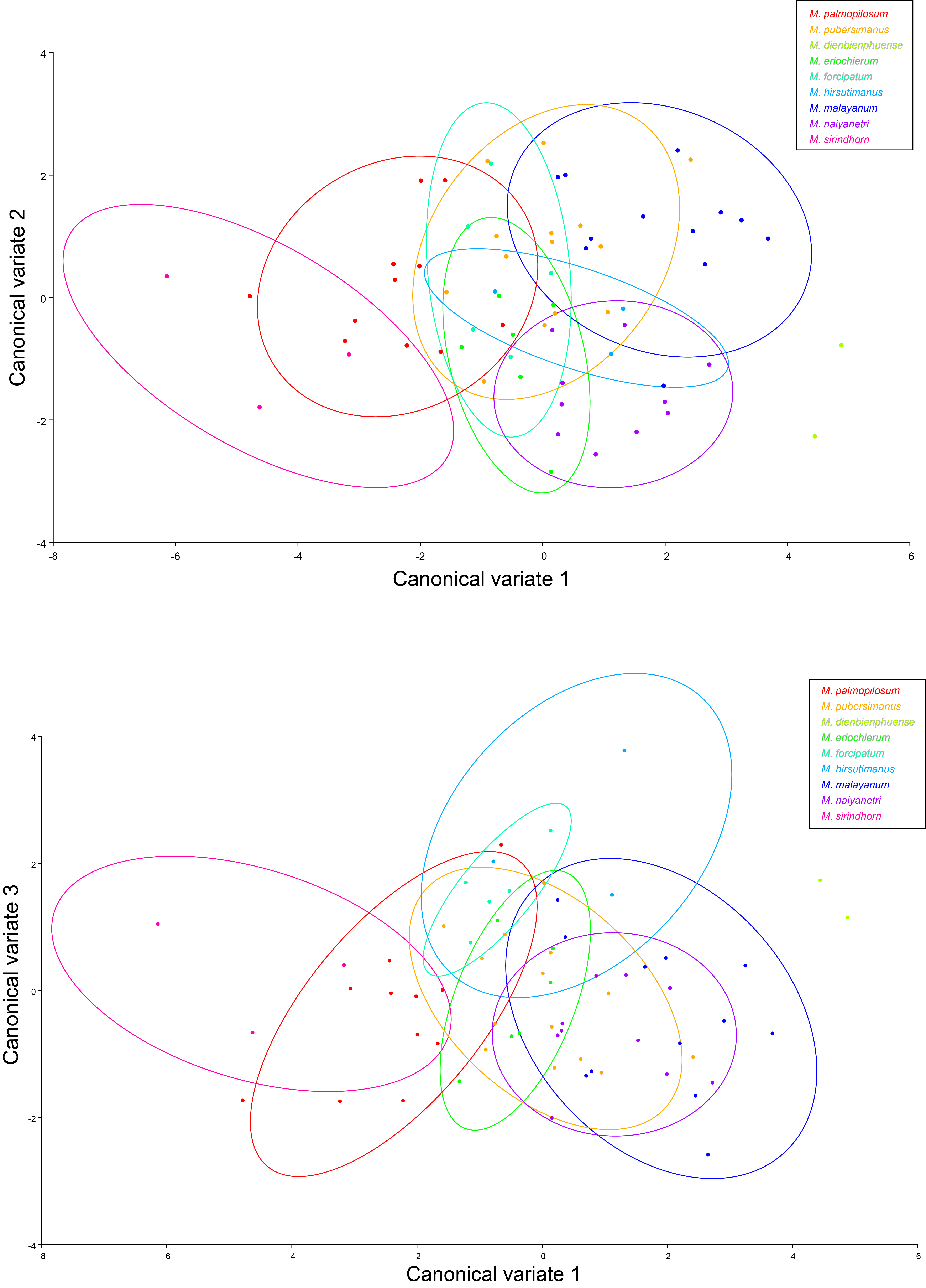

Supplement: Figure S5 [file peerj-08-10137-s005.jpg]
