# Supplementary material for: Molecular phylogeny and species delimitation of the freshwater prawn Macrobrachium pilimanus species group, with descriptions of three new species from Thailand
Source: PeerJ. 2020 Nov 27;8:e10137. doi: 10.7717/peerj.10137 (PMC7703394; doi:10.7717/peerj.10137)
Supplement: Figure S2 [file peerj-08-10137-s002.pdf]

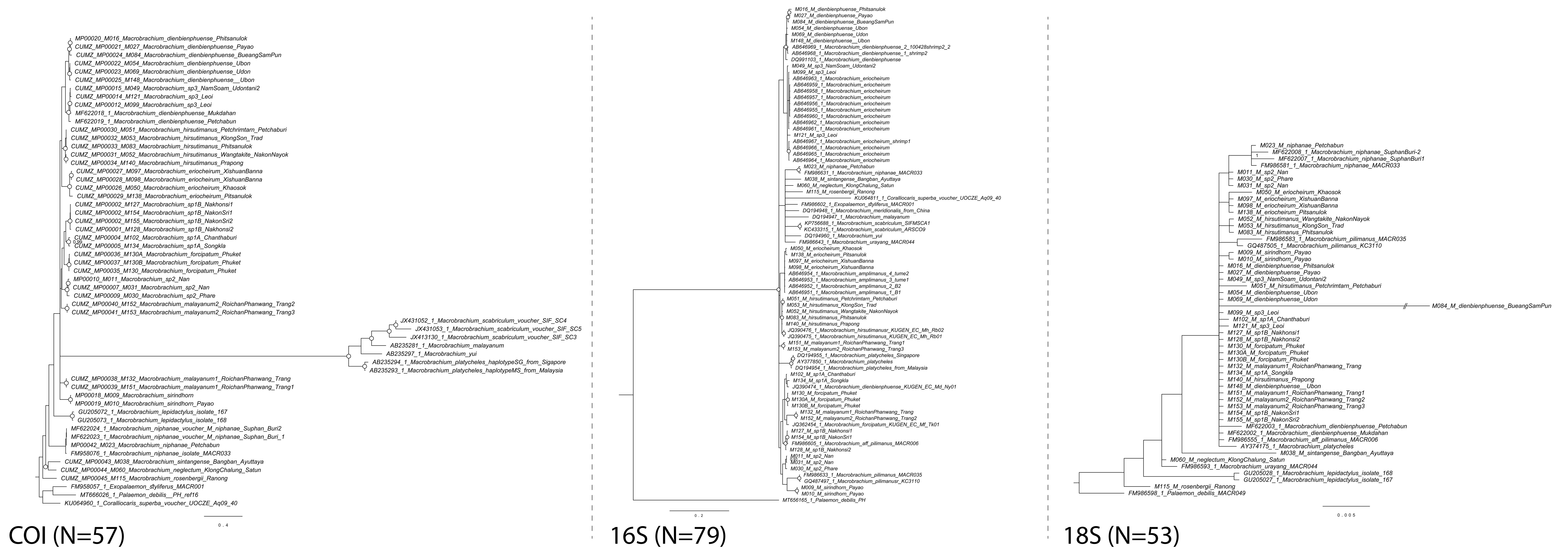
MP00020_M016_Macrobrachium_dienbienphuense_Phitsanulok
CUMZ_MP00021_M027_Macrobrachium_dienbienphuense_Payao
CUMZ_MP00024_M084_Macrobrachium_dienbienphuense_BueangSamPun
CUMZ_MP00022_M054_Macrobrachium_dienbienphuense_Ubon
CUMZ_MP00023_M069_Macrobrachium_dienbienphuense_Udon
CUMZ_MP00025_M148_Macrobrachium_dienbienphuense__Ubon
CUMZ_MP00015_M049_Macrobrachium_sp3_NamSoam_Udontani2
CUMZ_MP00014_M121_Macrobrachium_sp3_Leoi
CUMZ_MP00012_M099_Macrobrachium_sp3_Leoi
MF622018_1_Macrobrachium_dienbienphuense_Mukdahan
MF622019_1_Macrobrachium_dienbienphuense_Petchabun
CUMZ_MP00030_M051_Macrobrachium_hirsutimanus_Petchrimtarn_Petchaburi
CUMZ_MP00032_M053_Macrobrachium_hirsutimanus_KlongSon_Trad
CUMZ_MP00033_M083_Macrobrachium_hirsutimanus_Phitsanulok
CUMZ_MP00031_M052_Macrobrachium_hirsutimanus_Wangtakite_NakonNayok
CUMZ_MP00034_M140_Macrobrachium_hirsutimanus_Prapong
CUMZ_MP00027_M097_Macrobrachium_eriocheirum_XishuanBanna
CUMZ_MP00028_M098_Macrobrachium_eriocheirum_XishuanBanna
CUMZ_MP00026_M050_Macrobrachium_eriocheirum_Khaosok
CUMZ_MP00029_M138_Macrobrachium_eriocheirum_Pitsanulok
CUMZ_MP00002_M127_Macrobrachium_sp1B_Nakhonsi1
CUMZ_MP00002_M154_Macrobrachium_sp1B_NakonSri1
CUMZ_MP00002_M155_Macrobrachium_sp1B_NakonSri2
CUMZ_MP00001_M128_Macrobrachium_sp1B_Nakhonsi2
CUMZ_MP00004_M102_Macrobrachium_sp1A_Chanthaburi
0.99
CUMZ_MP00005_M134_Macrobrachium_sp1A_Songkla
CUMZ_MP00036_M130A_Macrobrachium_forcipatum_Phuket
CUMZ_MP00037_M130B_Macrobrachium_forcipatum_Phuket
CUMZ_MP00035_M130_Macrobrachium_forcipatum_Phuket
MP00010_M011_Macrobrachium_sp2_Nan
CUMZ_MP00007_M031_Macrobrachium_sp2_Nan
CUMZ_MP00009_M030_Macrobrachium_sp2_Phare
CUMZ_MP00040_M152_Macrobrachium_malayanum2_RoichanPhanwang_Trang2
CUMZ_MP00041_M153_Macrobrachium_malayanum2_RoichanPhanwang_Trang3
JX431052_1_Macrobrachium_scabriculum_voucher_SIF_SC4
JX431053_1_Macrobrachium_scabriculum_voucher_SIF_SC5
JX413130_1_Macrobrachium_scabriculum_voucher_SIF_SC3
AB235281_1_Macrobrachium_malayanum
AB235297_1_Macrobrachium_yui
AB235294_1_Macrobrachium_platycheles_haplotypeSG_from_Sigapore
AB235293_1_Macrobrachium_platycheles_haplotypeMS_from_Malaysia
CUMZ_MP00038_M132_Macrobrachium_malayanum1_RoichanPhanwang_Trang
CUMZ_MP00039_M151_Macrobrachium_malayanum1_RoichanPhanwang_Trang1
MP00018_M009_Macrobrachium_sirindhorn
MP00019_M010_Macrobrachium_sirindhorn_Payao
GU205072_1_Macrobrachium_lepidactylus_isolate_167
GU205073_1_Macrobrachium_lepidactylus_isolate_168
MF622024_1_Macrobrachium_niphanae_voucher_M_niphanae_Suphan_Buri2
MF622023_1_Macrobrachium_niphanae_voucher_M_niphanae_Suphan_Buri_1
MP00042_M023_Macrobrachium_niphanae_Petchabun
FM958076_1_Macrobrachium_niphanae_isolate_MACR033
CUMZ_MP00043_M038_Macrobrachium_sintangense_Bangban_Ayuttaya
CUMZ_MP00044_M060_Macrobrachium_neglectum_KlongChalung_Satun
CUMZ_MP00045_M115_Macrobrachium_rosenbergii_Ranong
FM958057_1_Exopalaemon_styliferus_MACR001
MT666026_1_Palaemon_debilis__PH_ref16
KU064960_1_Coralliocaris_superba_voucher_UOCZE_Aq09_40
0.4
COI (N=57)
M016_M_dienbienphuense_Phitsanulok
M027_M_dienbienphuense_Payao
M084_M_dienbienphuense_BueangSamPun
M054_M_dienbienphuense_Ubon
M069_M_dienbienphuense_Udon
M148_M_dienbienphuense__Ubon
AB646969_1_Macrobrachium_dienbienphuense_2_100428shrimp2_2
AB646968_1_Macrobrachium_dienbienphuense_1_shrimp2
DQ991103_1_Macrobrachium_dienbienphuense
M049_M_sp3_NamSoam_Udontani2
M099_M_sp3_Leoi
AB646963_1_Macrobrachium_eriocheirum
AB646959_1_Macrobrachium_eriocheirum
AB646958_1_Macrobrachium_eriocheirum
AB646957_1_Macrobrachium_eriocheirum
AB646956_1_Macrobrachium_eriocheirum
AB646955_1_Macrobrachium_eriocheirum
AB646960_1_Macrobrachium_eriocheirum
AB646962_1_Macrobrachium_eriocheirum
AB646961_1_Macrobrachium_eriocheirum
M121_M_sp3_Leoi
AB646967_1_Macrobrachium_eriocheirum_shrimp1
AB646966_1_Macrobrachium_eriocheirum
AB646965_1_Macrobrachium_eriocheirum
AB646964_1_Macrobrachium_eriocheirum
M023_M_niphanae_Petchabun
FM986631_1_Macrobrachium_niphanae_MACR033
M038_M_sintangense_Bangban_Ayuttaya
M060_M_neglectum_KlongChalung_Satun
M115_M_rosenbergii_Ranong
KU064811_1_Coralliocaris_superba_voucher_UOCZE_Aq09_40
FM986602_1_Exopalaemon_styliferus_MACR001
DQ194948_1_Macrobrachium_meridionalis_from_China
DQ194947_1_Macrobrachium_malayanum
KP756688_1_Macrobrachium_scabriculum_SIFMSCA1
KC433315_1_Macrobrachium_scabriculum_ARSCO9
DQ194960_1_Macrobrachium_yui
FM986643_1_Macrobrachium_urayang_MACR044
M050_M_eriocheirum_Khaosok
M138_M_eriocheirum_Pitsanulok
M097_M_eriocheirum_XishuanBanna
M098_M_eriocheirum_XishuanBanna
AB646954_1_Macrobrachium_amplimanus_4_tume2
AB646953_1_Macrobrachium_amplimanus_3_tume1
AB646952_1_Macrobrachium_amplimanus_2_B2
AB646951_1_Macrobrachium_amplimanus_1_B1
M051_M_hirsutimanus_Petchrimtarn_Petchaburi
M053_M_hirsutimanus_KlongSon_Trad
M052_M_hirsutimanus_Wangtakite_NakonNayok
M083_M_hirsutimanus_Phitsanulok
M140_M_hirsutimanus_Prapong
JQ390476_1_Macrobrachium_hirsutimanusr_KUGEN_EC_Mh_Rb02
JQ390475_1_Macrobrachium_hirsutimanus_KUGEN_EC_Mh_Rb01
M151_M_malayanum1_RoichanPhanwang_Trang1
M153_M_malayanum2_RoichanPhanwang_Trang3
DQ194955_1_Macrobrachium_platycheles_Singapore
AY377850_1_Macrobrachium_platycheles
DQ194954_1_Macrobrachium_platycheles_from_Malaysia
M102_M_sp1A_Chanthaburi
M134_M_sp1A_Songkla
JQ390474_1_Macrobrachium_dienbienphuense_KUGEN_EC_Md_Ny01
M130_M_forcipatum_Phuket
M130A_M_forcipatum_Phuket
M130B_M_forcipatum_Phuket
M132_M_malayanum1_RoichanPhanwang_Trang
M152_M_malayanum2_RoichanPhanwang_Trang2
JQ362454_1_Macrobrachium_forcipatum_KUGEN_EC_Mf_Tk01
M127_M_sp1B_Nakhonsi1
M154_M_sp1B_NakonSri1
FM986605_1_Macrobrachium_aff_pilimanus_MACR006
M128_M_sp1B_Nakhonsi2
M011_M_sp2_Nan
M031_M_sp2_Nan
M030_M_sp2_Phare
FM986633_1_Macrobrachium_pilimanus_MACR035
GQ487497_1_Macrobrachium_pilimanusr_KC3110
M009_M_sirindhorn_Payao
M010_M_sirindhorn_Payao
MT656165_1_Palaemon_debilis_PH
0.2
16S (N=79)
M023_M_niphanae_Petchabun
MF622008_1_Macrobrachium_niphanae_SuphanBuri-2
1
MF622007_1_Macrobrachium_niphanae_SuphanBuri1
FM986581_1_Macrobrachium_niphanae_MACR033
M011_M_sp2_Nan
M030_M_sp2_Phare
M031_M_sp2_Nan
M050_M_eriocheirum_Khaosok
M097_M_eriocheirum_XishuanBanna
M098_M_eriocheirum_XishuanBanna
M138_M_eriocheirum_Pitsanulok
M052_M_hirsutimanus_Wangtakite_NakonNayok
M053_M_hirsutimanus_KlongSon_Trad
M083_M_hirsutimanus_Phitsanulok
FM986583_1_Macrobrachium_pilimanus_MACR035
GQ487505_1_Macrobrachium_pilimanus_KC3110
M009_M_sirindhorn_Payao
M010_M_sirindhorn_Payao
M016_M_dienbienphuense_Phitsanulok
M027_M_dienbienphuense_Payao
M049_M_sp3_NamSoam_Udontani2
M051_M_hirsutimanus_Petchrimtarn_Petchaburi
M054_M_dienbienphuense_Ubon
M069_M_dienbienphuense_Udon
M084_M_dienbienphuense_BueangSamPun
M099_M_sp3_Leoi
M102_M_sp1A_Chanthaburi
M121_M_sp3_Leoi
M127_M_sp1B_Nakhonsi1
M128_M_sp1B_Nakhonsi2
M130_M_forcipatum_Phuket
M130A_M_forcipatum_Phuket
M130B_M_forcipatum_Phuket
M132_M_malayanum1_RoichanPhanwang_Trang
M134_M_sp1A_Songkla
M140_M_hirsutimanus_Prapong
M148_M_dienbienphuense__Ubon
M151_M_malayanum1_RoichanPhanwang_Trang1
M152_M_malayanum2_RoichanPhanwang_Trang2
M153_M_malayanum2_RoichanPhanwang_Trang3
M154_M_sp1B_NakonSri1
M155_M_sp1B_NakonSri2
MF622003_1_Macrobrachium_dienbienphuense_Petchabun
MF622002_1_Macrobrachium_dienbienphuense_Mukdahan
FM986555_1_Macrobrachium_aff_pilimanus_MACR006
AY374175_1_Macrobrachium_platycheles
M038_M_sintangense_Bangban_Ayuttaya
M060_M_neglectum_KlongChalung_Satun
FM986593_1_Macrobrachium_urayang_MACR044
GU205028_1_Macrobrachium_lepidactylus_isolate_168
GU205027_1_Macrobrachium_lepidactylus_isolate_167
M115_M_rosenbergii_Ranong
FM986598_1_Palaemon_debilis_MACR049
0.005
18S (N=53)
