# Supplementary material for: Molecular phylogeny and species delimitation of the freshwater prawn Macrobrachium pilimanus species group, with descriptions of three new species from Thailand
Source: PeerJ. 2020 Nov 27;8:e10137. doi: 10.7717/peerj.10137 (PMC7703394; doi:10.7717/peerj.10137)
Supplement: Table S1 [file peerj-08-10137-s006.docx]

**Table S1.** **The type locality and distribution range of members in *M. pilimanus* group**

| Species | Type locality | Distribution | References |
| --- | --- | --- | --- |
| *M. ahkowi* Chong and Khoo, 1987 | Gunong Palai, Johore, Malaysia | Only known from Type locality and water tributary nearby | Chong and Khoo, 1987 |
| *M. amplimanus* Cai & Dai,1999 | Mengla County, Yunan, China | **China:** Yunan  **Laos:** Luang Prabang  **Thailand:** Chiang Mai, Mekong river basin, Kanchanaburi, Narathiwat | Cai & Dai, 1999; Cai et al. 2004; Hanamura et al. 2011 |
| *M. dalatense* Xuan, 2003 | Krean, Lam Dong, Dalat, South Vietnam | Only known from Type locality and water tributary nearby | Xuan, 2003 |
| *M. dienbienphuense* Dany & Nguyen, 1972 | Song Nam Rom, Dien Bien Phu, North Vietnam | **China:** Yunnan  **Vietnam:** Dien Bien Phu  **Laos:** Luang Prabang  **Thailand:** Chiang Mai, Nan, Chiang Rai, Saraburi, Lopburi**,** Ubon Ratchathani, Nakhon Phanom, Loei, Nong Bua Lam Pu, Nakhon Ratchatsima, Chantaburi, Trat**,** Krabi, Narathiwat, Phatthalung | Dany & Nguyen, 1972; Cai et al. 2004;  Hanamura et al. 2011 |
| *M. empulipke* Wowor, 2010 | Sugai Cibogo, Sukabumi Regency,West Java, Indonesia | **Java:** Bogor, Cigudeg, Banten **Sumatra:** Lampung, Danau Dibawah, Danau Diatas, Lake Singkarak | Wowor, 2010 |
| *M. eriocheirum* Dai, 1984 | Jingsan, Yunnan, China | **China:** Yunan  **Laos:** Luang Prabang  **Thailand:** Lampang, Uthaitani**,** Tak**,** Ubon Ratchathani**,** Chantaburi, Trat | Cai et al. 2004; Hanamura et al. 2011 |
| *M. forcipatum* Ng, 1995 | Tasik Temengor, south of Banding, mouth of Sungai Halong, Perak, Malaysia | **Malaysia:** Perak  **Thailand:** Ratchaburi, Krabi, Narathiwat, Phuket, Songkhla, Phatthalung, Chumpon, Satun, Nakhon Si Thammarat | Ng, 1995; Cai et al. 2004; Hanamura et al. 2011 |
| *M. gua* Chong, 1989 | Gomantong Hill, Sabah, Borneo | Only known from Type locality and water tributary nearby | Chong, 1989 |
| *M. hirsutimanus* (Tiwari, 1952) | forest stream 95km north from Nan Town, Nan, Thailand | **Thailand:** Nan, Chiang Mai, Uttaradit, Phrae, Lampang, Uthaitani**,** Tak**,** Saraburi**,** Nakhon Ratchatsima, Maha Sarakham, Ubon Ratchathani**,** Chantaburi, Trat | Cai et al. 2004; Hanamura et al. 2011 |
| *M. kelianense* Wowor and Short, 2007 | Sigai Kelian confluence with Sugai Ketang Mahakam River  basin, East Kalimantan, Indonesia | Only known from Type locality and water tributary nearby | Wowor and Short, 2007 |
| *M. leptodactylus* (De Man, 1892) | Bogor, Java | Only known from Type locality | De Man, 1892 |
| *M. malayanum* (Roux, 1934) | Lasah, Plus Valley,  Perak, Malay Peninsula (lectotype designation) | **Malaysia:** Johore, Malacca, Pahang, Selangor, Perak  **Borneo:** Sarawak  **Singapore:**  Bukit Timah Nature Reserve, Nee Soon  **Sumatra:** Jambi  **Thailand:** Narathiwat**,** Trang | Hanamura et al. 201 |
| *M. pilimanus* (De Man, 1879) | Moearalaboeh, West Sumatra, Indonesia (lectotype designation) | **Sumatra:** Alahanpanjang, Manindjau Lake, Singkarak Lake, Bua Cave, Andala, Muaralabih, Medan, Bataklanden, Petok, Manindjau, Fort de Kock, Kota Bharu, Anai Canyon, Singkarak Lake, Sidjundjung, Danau di Atas, River Selangis, Mt. Dempo, Korinchi Lake **Malaysia:** Perlis, Kelantan, Pahang, Perak, Johore, Sarawak  **Singapore:** Nee Soon  **Borneo:** Sanggau, Sintang, Ketungau **Java:** Sinagar, Bogor, Situ Bagendit, Situ Cibodas, Tjibabalukan, Tretes, Malang, Sukabumi, Tjinjiruan; Pasirdatar; Ungaran Mountain; Mendit, Bawean **Vietnam:** no exact locality record | De Man, 1892; Johnson, 1961; Dang, 1998 |
| *M. pilosum* Cai and Dai, 1999 | Mengla County, Yunan, China | Only known from Type locality and water tributary nearby | Cai & Dai,1999 |
| *M. platycheles* Ou and Yeo, 1995 | Nee Soon swamp forest, Singapore | **Singapore:** Nee Soon swamp forest, Lorong Banir  **Malaysia:** Johore, Malacca | Ou and Yeo, 1995 |
| *M. sirindhorn* Naiyanetr, 2001 | Pong Nam Dung Waterfall, Mae Soon, Fang, Chiang Mai, Thailand | **Thailand:** Chiang Mai, Phayao,  Mae Hong Son | Naiyanetr, et al. 2001; Cai et al. 2004; |
| *M. spelaeus* Cai and Vidthayanon, 2016 | Tham Phra Wangdaeng, Thung Salaeng Luang National Park, Pitsanulok, Thailand | Only known from Type locality | Cai and Vidthayanon, 2016 |
| *M. urayang* Wowor and Short, 2007 | Sugai Bahau, Kayan basin, East Kalimantan, Indonesia | Only known from Type locality and water tributary nearby | Wowor and Short, 2007 |
