# Supplementary material for: Molecular phylogeny and species delimitation of the freshwater prawn Macrobrachium pilimanus species group, with descriptions of three new species from Thailand
Source: PeerJ. 2020 Nov 27;8:e10137. doi: 10.7717/peerj.10137 (PMC7703394; doi:10.7717/peerj.10137)
Supplement: Table S2 [file peerj-08-10137-s007.docx]

**Table S2.** GenBank accession numbers of additional sequences including outgroup taxa used in this study (n/a is data not provided in reference literature or database)

| **Species** | **Locality** | **Sequence accession number** | | |
| --- | --- | --- | --- | --- |
|  |  | **COI** | **16S** | **18S** |
| *M. platycheles* | Singapore | - | AY377850_1 | - |
|  | Malaysia | AB235293_1 | DQ194954_1 | - |
|  | Singapore | AB235294_1 | DQ194955_1 | - |
|  | n/a | - | - | AY374175_1 |
| *M. urayang* | East Kalimantan, Indonesia | - | FM986643_1 | FM986593_1 |
| *M. yui* | Yunnan, China | AB235297_1 | DQ194960_1 | - |
| *Palaemon debilis* | n/a | MT666026_1 | MT656165_1 | FM986598_1 |
| *M. scabriculum* | n/a | - | KC433315_1 | - |
|  | India | JX431053_1 | KP756688_1 | - |
|  | n/a | JX413130_1 | - | - |
|  | n/a | JX431052_1 | - | - |
| *M. aff. pilimanus* | Khammouan, Laos | - | FM986605_1 | FM986555_1 |
| *M. pilimanus* | n/a | - | GQ487497_1 | GQ487505_1 |
|  | Tioman, Malaysia | - | FM986633_1 | FM986583_1 |
| *M. malayanum* | Gunonghedang, Malaysia | AB235281_1 | DQ194947_1 | - |
| *M. meridionalis* | Hainan, China | - | DQ194948_1 | - |
| *M. eriocheirum* | Luang Prabang, Laos | - | AB646960_1 | - |
|  | Luang Prabang, Laos | - | AB646961_1 | - |
|  | Luang Prabang, Laos | - | AB646962_1 | - |
|  | Luang Prabang, Laos | - | AB646955_1 | - |
|  | Luang Prabang, Laos | - | AB646956_1 | - |
|  | Luang Prabang, Laos | - | AB646957_1 | - |
|  | Luang Prabang, Laos | - | AB646958_1 | - |
|  | Luang Prabang, Laos | - | AB646959_1 | - |
|  | Luang Prabang, Laos | - | AB646963_1 | - |
|  | Luang Prabang, Laos | - | AB646964_1 | - |
|  | Luang Prabang, Laos | - | AB646965_1 | - |
|  | Luang Prabang, Laos | - | AB646966_1 | - |
|  | Luang Prabang, Laos | - | AB646967_1 | - |
| *M. dienbienphuense* | Luang Prabang, Laos | - | AB646968_1 | - |
|  | Luang Prabang, Laos | - | AB646969_1 | - |
|  | n/a | - | JQ390474_1 | - |
|  | n/a | - | DQ991103_1 | - |
|  | Mukdahan, Thailand | MF622018_1 | - | MF622002_1 |
|  | Phetchabun, Thailand | MF622019_1 | - | MF622003_1 |
| *M. niphanae* | Perlis, Malaysia | FM958076_1 | FM986631_1 | FM986581_1 |
|  | Suphanburi, Thailand | MF622023_1 | - | MF622007_1 |
|  | Suphanburi, Thailand | MF622024_1 | - | MF622008_1 |
| *M. lepidactylus* | n/a | GU205073_1 | - | GU205028_1 |
|  | n/a | GU205072_1 | - | GU205027_1 |
| *M. hirsutimanus* | n/a | - | JQ390475_1 | - |
|  | n/a | - | JQ390476_1 | - |
| *M. forcipatum* | n/a | - | JQ362454_1 | - |
| *M. amplimanus* | Luang Prabang, Laos | - | AB646951_1 | - |
|  | Luang Prabang, Laos | - | AB646952_1 | - |
|  | Luang Prabang, Laos | - | AB646953_1 | - |
|  | Luang Prabang, Laos | - | AB646954_1 | - |
| *Exopalaemon styliferus* | Tioman, Malaysia | FM958057_1 | FM986602_1 | - |
| *Coralliocaris superba* | Jordan | KU064960_1 | KU064811_1 | - |
