# Supplementary material for: Molecular phylogeny and species delimitation of the freshwater prawn Macrobrachium pilimanus species group, with descriptions of three new species from Thailand
Source: PeerJ. 2020 Nov 27;8:e10137. doi: 10.7717/peerj.10137 (PMC7703394; doi:10.7717/peerj.10137)
Supplement: Table S3 — The blue text indicates standard deviation. [file peerj-08-10137-s008.docx]

| \| species \| genetic distance / standard deviation \| \| \| \| \| \| \| \| \| \| \| \| \| \| --- \| --- \| --- \| --- \| --- \| --- \| --- \| --- \| --- \| --- \| --- \| --- \| --- \| --- \| \| *M. sirinhorn* \|  \| 0.020 \| 0.020 \| 0.029 \| 0.026 \| 0.021 \| 0.024 \| 0.020 \| 0.025 \| 0.019 \| 0.026 \| 0.021 \| 0.019 \| \| *M. palmopilosum* \| 0.178 \|  \| 0.018 \| 0.025 \| 0.024 \| 0.020 \| 0.018 \| 0.015 \| 0.023 \| 0.014 \| 0.024 \| 0.015 \| 0.014 \| \| *M. dienbienphuense* \| 0.184 \| 0.153 \|  \| 0.028 \| 0.026 \| 0.013 \| 0.018 \| 0.015 \| 0.023 \| 0.015 \| 0.024 \| 0.017 \| 0.015 \| \| *M. niphanae* \| 0.279 \| 0.238 \| 0.278 \|  \| 0.025 \| 0.027 \| 0.027 \| 0.026 \| 0.023 \| 0.024 \| 0.026 \| 0.026 \| 0.026 \| \| *M. sintangense* \| 0.232 \| 0.221 \| 0.242 \| 0.232 \|  \| 0.028 \| 0.025 \| 0.024 \| 0.019 \| 0.024 \| 0.025 \| 0.024 \| 0.024 \| \| *M. puberimanus* \| 0.182 \| 0.168 \| 0.098 \| 0.270 \| 0.264 \|  \| 0.018 \| 0.016 \| 0.023 \| 0.017 \| 0.024 \| 0.017 \| 0.017 \| \| *M. eriocheirum* \| 0.233 \| 0.166 \| 0.163 \| 0.263 \| 0.233 \| 0.152 \|  \| 0.015 \| 0.024 \| 0.017 \| 0.026 \| 0.019 \| 0.017 \| \| *M. hirsutimanus* \| 0.173 \| 0.127 \| 0.128 \| 0.239 \| 0.227 \| 0.125 \| 0.125 \|  \| 0.022 \| 0.013 \| 0.025 \| 0.016 \| 0.014 \| \| *M. neglectum* \| 0.232 \| 0.225 \| 0.221 \| 0.222 \| 0.182 \| 0.216 \| 0.245 \| 0.209 \|  \| 0.022 \| 0.022 \| 0.024 \| 0.024 \| \| *M. naiyanetri* \| 0.169 \| 0.124 \| 0.142 \| 0.235 \| 0.222 \| 0.150 \| 0.157 \| 0.117 \| 0.219 \|  \| 0.023 \| 0.013 \| 0.014 \| \| *M. rosenbergii* \| 0.246 \| 0.225 \| 0.235 \| 0.253 \| 0.248 \| 0.228 \| 0.263 \| 0.229 \| 0.200 \| 0.229 \|  \| 0.025 \| 0.024 \| \| *M. forcipatum* \| 0.181 \| 0.122 \| 0.138 \| 0.249 \| 0.221 \| 0.135 \| 0.178 \| 0.132 \| 0.239 \| 0.101 \| 0.241 \|  \| 0.014 \| \| *M. malayanum* \| 0.183 \| 0.127 \| 0.137 \| 0.258 \| 0.245 \| 0.151 \| 0.166 \| 0.122 \| 0.249 \| 0.130 \| 0.242 \| 0.131 \|  \|   Table S3. **The interspecific variation of DNA sequence in COI gene** (the blue text indicates standard deviation) |  |  |  |  |  |  |  |  |  |  |  |  |  |
| --- | --- | --- | --- | --- | --- | --- | --- | --- | --- | --- | --- | --- | --- | --- | --- | --- | --- | --- | --- | --- | --- | --- | --- | --- | --- | --- | --- | --- | --- | --- | --- | --- | --- | --- | --- | --- | --- | --- | --- | --- | --- | --- | --- | --- | --- | --- | --- | --- | --- | --- | --- | --- | --- | --- | --- | --- | --- | --- | --- | --- | --- | --- | --- | --- | --- | --- | --- | --- | --- | --- | --- | --- | --- | --- | --- | --- | --- | --- | --- | --- | --- | --- | --- | --- | --- | --- | --- | --- | --- | --- | --- | --- | --- | --- | --- | --- | --- | --- | --- | --- | --- | --- | --- | --- | --- | --- | --- | --- | --- | --- | --- | --- | --- | --- | --- | --- | --- | --- | --- | --- | --- | --- | --- | --- | --- | --- | --- | --- | --- | --- | --- | --- | --- | --- | --- | --- | --- | --- | --- | --- | --- | --- | --- | --- | --- | --- | --- | --- | --- | --- | --- | --- | --- | --- | --- | --- | --- | --- | --- | --- | --- | --- | --- | --- | --- | --- | --- | --- | --- | --- | --- | --- | --- | --- | --- | --- | --- | --- | --- | --- | --- | --- | --- | --- | --- | --- | --- | --- | --- | --- | --- | --- | --- | --- | --- | --- | --- | --- | --- | --- | --- | --- | --- | --- | --- | --- | --- | --- | --- |
|  |  |  |  |  |  |  |  |  |  |  |  |  |  |
|  |  |  |  |  |  |  |  |  |  |  |  |  |  |
| Table S1. **The interspecific variation of DNA sequence in 16S rRNA gene** (the blue text indicates standard deviation) |  |  |  |  |  |  |  |  |  |  |  |  |  |
| \| *M. sirinhorn* \|  \| 0.011 \| 0.012 \| 0.016 \| 0.015 \| 0.012 \| 0.011 \| 0.011 \| 0.013 \| 0.011 \| 0.017 \| 0.012 \| 0.011 \| \| --- \| --- \| --- \| --- \| --- \| --- \| --- \| --- \| --- \| --- \| --- \| --- \| --- \| --- \| \| *M. palmopilosum* \| 0.062 \|  \| 0.009 \| 0.015 \| 0.016 \| 0.010 \| 0.010 \| 0.009 \| 0.013 \| 0.007 \| 0.014 \| 0.007 \| 0.008 \| \| *M. dienbienphuense* \| 0.072 \| 0.050 \|  \| 0.014 \| 0.017 \| 0.007 \| 0.009 \| 0.008 \| 0.014 \| 0.010 \| 0.015 \| 0.010 \| 0.009 \| \| *M. niphanae* \| 0.108 \| 0.095 \| 0.095 \|  \| 0.017 \| 0.016 \| 0.014 \| 0.013 \| 0.012 \| 0.014 \| 0.017 \| 0.014 \| 0.014 \| \| *M. sintangense* \| 0.108 \| 0.110 \| 0.120 \| 0.109 \|  \| 0.017 \| 0.016 \| 0.016 \| 0.013 \| 0.016 \| 0.019 \| 0.017 \| 0.017 \| \| *M. puberimanus* \| 0.077 \| 0.056 \| 0.033 \| 0.107 \| 0.126 \|  \| 0.009 \| 0.008 \| 0.014 \| 0.010 \| 0.016 \| 0.010 \| 0.010 \| \| *M. eriocheirum* \| 0.061 \| 0.053 \| 0.050 \| 0.084 \| 0.107 \| 0.048 \|  \| 0.006 \| 0.011 \| 0.010 \| 0.015 \| 0.010 \| 0.008 \| \| *M. hirsutimanus* \| 0.059 \| 0.040 \| 0.038 \| 0.074 \| 0.108 \| 0.040 \| 0.023 \|  \| 0.011 \| 0.008 \| 0.014 \| 0.009 \| 0.008 \| \| *M. neglectum* \| 0.082 \| 0.073 \| 0.087 \| 0.073 \| 0.083 \| 0.088 \| 0.058 \| 0.054 \|  \| 0.012 \| 0.014 \| 0.012 \| 0.012 \| \| *M. naiyanetri* \| 0.066 \| 0.032 \| 0.062 \| 0.090 \| 0.113 \| 0.063 \| 0.053 \| 0.044 \| 0.071 \|  \| 0.014 \| 0.006 \| 0.008 \| \| *M. rosenbergii* \| 0.118 \| 0.087 \| 0.100 \| 0.117 \| 0.141 \| 0.106 \| 0.093 \| 0.089 \| 0.092 \| 0.088 \|  \| 0.014 \| 0.014 \| \| *M. forcipatum* \| 0.074 \| 0.030 \| 0.054 \| 0.094 \| 0.125 \| 0.056 \| 0.052 \| 0.041 \| 0.069 \| 0.024 \| 0.090 \|  \| 0.008 \| \| *M. malayanum* \| 0.071 \| 0.047 \| 0.061 \| 0.100 \| 0.121 \| 0.068 \| 0.050 \| 0.045 \| 0.073 \| 0.045 \| 0.095 \| 0.043 \|  \| |  |  |  |  |  |  |  |  |  |  |  |  |  |
|  |  |  |  |  |  |  |  |  |  |  |  |  |  |
|  |  |  |  |  |  |  |  |  |  |  |  |  |  |
|  |  |  |  |  |  |  |  |  |  |  |  |  |  |
|  |  |  |  |  |  |  |  |  |  |  |  |  |  |
| Table S1. **The interspecific variation of DNA sequence in 18S rRNA gene** (the blue text indicates standard deviation) |  |  |  |  |  |  |  |  |  |  |  |  |  |
| \| species \| genetic distance \| \| \| \| \| \| \| \| \| \| \| \| \| \| --- \| --- \| --- \| --- \| --- \| --- \| --- \| --- \| --- \| --- \| --- \| --- \| --- \| --- \| \| *M. sirinhorn* \|  \| 0.001 \| 0.002 \| 0.002 \| 0.005 \| 0.001 \| 0.002 \| 0.001 \| 0.004 \| 0.001 \| 0.004 \| 0.001 \| 0.001 \| \| *M. palmopilosum* \| 0.002 \|  \| 0.002 \| 0.002 \| 0.005 \| 0.001 \| 0.001 \| 0.001 \| 0.003 \| 0.001 \| 0.004 \| 0.001 \| 0.001 \| \| *M. dienbienphuense* \| 0.011 \| 0.011 \|  \| 0.002 \| 0.005 \| 0.001 \| 0.002 \| 0.002 \| 0.003 \| 0.001 \| 0.004 \| 0.001 \| 0.002 \| \| *M. niphanae* \| 0.005 \| 0.003 \| 0.014 \|  \| 0.005 \| 0.002 \| 0.002 \| 0.002 \| 0.004 \| 0.002 \| 0.004 \| 0.002 \| 0.002 \| \| *M. sintangense* \| 0.024 \| 0.022 \| 0.032 \| 0.021 \|  \| 0.005 \| 0.005 \| 0.005 \| 0.004 \| 0.005 \| 0.005 \| 0.005 \| 0.005 \| \| *M. puberimanus* \| 0.001 \| 0.001 \| 0.011 \| 0.004 \| 0.023 \|  \| 0.002 \| 0.001 \| 0.003 \| 0.000 \| 0.004 \| 0.000 \| 0.001 \| \| *M. eriocheirum* \| 0.004 \| 0.002 \| 0.013 \| 0.005 \| 0.021 \| 0.003 \|  \| 0.002 \| 0.003 \| 0.002 \| 0.004 \| 0.002 \| 0.001 \| \| *M. hirsutimanus* \| 0.003 \| 0.003 \| 0.012 \| 0.006 \| 0.024 \| 0.002 \| 0.004 \|  \| 0.003 \| 0.001 \| 0.004 \| 0.001 \| 0.001 \| \| *M. neglectum* \| 0.012 \| 0.012 \| 0.020 \| 0.013 \| 0.018 \| 0.011 \| 0.012 \| 0.012 \|  \| 0.003 \| 0.003 \| 0.003 \| 0.003 \| \| *M. naiyanetri* \| 0.001 \| 0.001 \| 0.010 \| 0.004 \| 0.023 \| 0.000 \| 0.003 \| 0.002 \| 0.011 \|  \| 0.004 \| 0.000 \| 0.001 \| \| *M. rosenbergii* \| 0.018 \| 0.018 \| 0.026 \| 0.019 \| 0.024 \| 0.018 \| 0.018 \| 0.019 \| 0.009 \| 0.017 \|  \| 0.004 \| 0.004 \| \| *M. forcipatum* \| 0.001 \| 0.001 \| 0.010 \| 0.004 \| 0.023 \| 0.000 \| 0.003 \| 0.001 \| 0.011 \| 0.000 \| 0.017 \|  \| 0.001 \| \| *M. malayanum* \| 0.002 \| 0.002 \| 0.011 \| 0.005 \| 0.022 \| 0.001 \| 0.002 \| 0.003 \| 0.010 \| 0.001 \| 0.016 \| 0.001 \|  \| |  |  |  |  |  |  |  |  |  |  |  |  |  |
|  |  |  |  |  |  |  |  |  |  |  |  |  |  |
|  |  |  |  |  |  |  |  |  |  |  |  |  |  |

Table S1. **The intraspecific variation of DNA sequence in COI gene**

| Species | Genetic distance | Standard deviation |
| --- | --- | --- |
| *M. sirinhorn* | 0.004 | 0.003 |
| *M. palmopilosum* | 0.028 | 0.005 |
| *M. dienbienphuense* | 0.026 | 0.005 |
| *M. niphanae* | n/c | n/c |
| *M. sintangense* | n/c | n/c |
| *M. puberimanus* | 0.011 | 0.003 |
| *M. eriocheirum* | 0.040 | 0.006 |
| *M. hirsutimanus* | 0.011 | 0.003 |
| *M. neglectum* | n/c | n/c |
| *M. naiyanetri* | 0.046 | 0.006 |
| *M. rosenbergii* | n/c | n/c |
| *M. forcipatum* | 0.005 | 0.002 |
| *M. malayanum* | 0.084 | 0.011 |

Table S1. **The intraspecific variation of DNA sequence in 16S rRNA gene**

| Species | Genetic distance | Standard deviation |
| --- | --- | --- |
| *M. sirinhorn* | 0.002 | 0.002 |
| *M. palmopilosum* | 0.004 | 0.002 |
| *M. dienbienphuense* | 0.010 | 0.003 |
| *M. niphanae* | n/c | n/c |
| *M. sintangense* | n/c | n/c |
| *M. puberimanus* | 0.010 | 0.003 |
| *M. eriocheirum* | 0.005 | 0.002 |
| *M. hirsutimanus* | 0.002 | 0.001 |
| *M. neglectum* | n/c | n/c |
| *M. naiyanetri* | 0.012 | 0.003 |
| *M. rosenbergii* | n/c | n/c |
| *M. forcipatum* | 0 | 0 |
| *M. malayanum* | 0.035 | 0.007 |

Table S1. **The intraspecific variation of DNA sequence in 16S rRNA gene**

| Species | Genetic distance | Standard deviation |
| --- | --- | --- |
| *M. sirinhorn* | 0.000 | 0.000 |
| *M. palmopilosum* | 0.000 | 0.000 |
| *M. dienbienphuense* | 0.021 | 0.003 |
| *M. niphanae* | n/c | n/c |
| *M. sintangense* | n/c | n/c |
| *M. puberimanus* | 0.001 | 0.001 |
| *M. eriocheirum* | 0.002 | 0.001 |
| *M. hirsutimanus* | 0.002 | 0.001 |
| *M. neglectum* | n/c | n/c |
| *M. naiyanetri* | 0.000 | 0.000 |
| *M. rosenbergii* | n/c | n/c |
| *M. forcipatum* | 0.000 | 0.000 |
| *M. malayanum* | 0.000 | 0.000 |
