# Supplementary material for: Molecular phylogeny and species delimitation of the freshwater prawn Macrobrachium pilimanus species group, with descriptions of three new species from Thailand
Source: PeerJ. 2020 Nov 27;8:e10137. doi: 10.7717/peerj.10137 (PMC7703394; doi:10.7717/peerj.10137)
Supplement: Table S6 [file peerj-08-10137-s011.docx]

**Table S6** The result of Procrustes ANOVA analyses

| **Character** | **Centroid size** | | | **Shape** | | |
| --- | --- | --- | --- | --- | --- | --- |
|  | df | f | *P*-value | df | f | *P*-value |
| Rostrum+carapace | 8 | 5.13 | <0.001 | 128 | 2.89 | <0.001 |
|  |  |  |  |  |  |  |

**Note:** df-degrees of freedom, *F*- *F* statistic value and *p*- *p* statistical significance value (p-values below 0.0001 suggest no significant error from landmark acquisition in each analysis)
