# Supplementary material for: Molecular phylogeny and species delimitation of the freshwater prawn Macrobrachium pilimanus species group, with descriptions of three new species from Thailand
Source: PeerJ. 2020 Nov 27;8:e10137. doi: 10.7717/peerj.10137 (PMC7703394; doi:10.7717/peerj.10137)
Supplement: Table S7 [file peerj-08-10137-s012.docx]

**Table S7.1** P-values from permutation tests (10,000 permutation rounds) for Mahalanobis distances among species

| Species | *M. palmopilosum* | *M. pubersimanus* | *M. dienbienphuense* | *M. eriocheirum* | *M. forcipatum* | *M. hirsutimanus* | *M. malayanum* | *M. naiyanetri* |
| --- | --- | --- | --- | --- | --- | --- | --- | --- |
| *M. pubersimanus* | <0.0001 |  |  |  |  |  |  |  |
| *M. dienbienphuense* | 0.0007 | 0.0048 |  |  |  |  |  |  |
| *M. eriocheirum* | 0.0008 | 0.0009 | 0.0352 |  |  |  |  |  |
| *M. forcipatum* | 0.0054 | 0.0044 | <0.0001 | 0.0089 |  |  |  |  |
| *M. hirsutimanus* | 0.0046 | 0.0128 | <0.0001 | 0.0251 | 0.1167 |  |  |  |
| *M. malayanum* | <0.0001 | 0.0001 | 0.0198 | <0.0001 | 0.0001 | 0.0277 |  |  |
| *M. naiyanetri* | <0.0001 | <0.0001 | 0.0076 | 0.0052 | 0.0052 | 0.0104 | 0.0001 |  |
| *M. sirindhorn* | 0.0363 | 0.0007 | 0.1053 | 0.0026 | 0.0026 | 0.1016 | 0.0001 | 0.0024 |

**Table S7.2** The P-values from permutation tests (10,000 permutation rounds) for Procrustes distances among species

| Species | *M. palmopilosum* | *M. pubersimanus* | *M. dienbienphuense* | *M. eriocheirum* | *M. forcipatum* | *M. hirsutimanus* | *M. malayanum* | *M. naiyanetri* |
| --- | --- | --- | --- | --- | --- | --- | --- | --- |
| *M. pubersimanus* | 0.0018 |  |  |  |  |  |  |  |
| *M. dienbienphuense* | 0.0087 | 0.0046 |  |  |  |  |  |  |
| *M. eriocheirum* | 0.4084 | 0.0417 | 0.0048 |  |  |  |  |  |
| *M. forcipatum* | 0.0611 | 0.0667 | 0.0467 | 0.0735 |  |  |  |  |
| *M. hirsutimanus* | 0.0154 | 0.0238 | 0.1734 | 0.0691 | 0.2287 |  |  |  |
| *M. malayanum* | 0.0007 | 0.0230 | 0.5932 | 0.0308 | 0.2502 | 0.3220 |  |  |
| *M. naiyanetri* | <0.0001 | 0.0005 | 0.2778 | 0.0006 | 0.0070 | 0.1302 | 0.8437 |  |
| *M. sirindhorn* | 0.1078 | 0.0056 | 0.1053 | 0.0347 | 0.0208 | 0.1615 | 0.0406 | 0.0019 |
